# Sex differences in fibrillar collagen composition are linked to subcutaneous adipose tissue expandability and adipocyte precursor proliferation

**DOI:** 10.1186/s13293-026-01012-6

**Published:** 2026-11-13

**Authors:** Milica Vujičić, Isabella Broderick, Marco Bauzá-Thorbrügge, Charlène Perian, Gabriela Campanher Pereira, Per-Anders Jansson, Matthias Blüher, Anna Benrick, Ingrid Wernstedt Asterholm

**Affiliations:** 1 Unit for Metabolic Physiology, Department of Physiology, Institute of Neuroscience and Physiology, The Sahlgrenska Academy at the University of Gothenburg, Gothenburg, Sweden; 2 The Wallenberg Laboratory and Sahlgrenska Center for Cardiovascular and Metabolic Research, Department of Molecular and Clinical Medicine, Institute of Medicine, University of Gothenburg, Gothenburg, Sweden; 3 Helmholtz Institute for Metabolic, Obesity and Vascular Research of the Helmholtz Zentrum München at the University of Leipzig and University Hospital Leipzig, Leipzig, Germany; 4 Medical Department III - Endocrinology, Nephrology, Rheumatology, University of Leipzig Medical Center, Leipzig, Germany; 5 School of Health Sciences, University of Skövde, Skövde, Sweden

**Keywords:** Adipogenic stem cells, Adipose tissue, Collagen, Sex difference

## Abstract

**Background:**

Physiological expansion of subcutaneous adipose tissue (SAT) protects against harmful lipid deposition and metabolic disease and is generally greater in women than men. We hypothesised that sex differences in fibrillar collagen composition and remodelling contribute to differences in SAT expansion and function.

**Methods:**

SAT from age- and BMI-matched men and women, SAT from male and female mice exposed to a high-fat diet to induce adipose tissue expansion and obesity, and publicly available human SAT and VAT RNA sequencing data sets were analysed. SAT was assessed by proteomics, histology, Western blot, qRT-PCR and flow cytometry. The 3T3-L1 preadipocyte cell line was used to test the effect of altered collagen type 1-to-3 ratio.

**Results:**

Men showed greater SAT fibrosis than women. Fibrillar collagen levels correlated negatively with BMI in women but positively with cholesterol and liver enzyme levels in men. Despite similar adiposity, HFD-induced obese female mice had healthier metabolic profiles and fewer fibroinflammatory SAT changes than males. HFD preferentially induced *Col1a1* in males and *Col3a1* in females. Unchallenged male mice had a higher collagen type 1-to-3 ratio than females, and a similar sex difference was observed in humans. A lower, female-like collagen type 1-to-3 ratio increased preadipocyte proliferation. Consistently, acute HFD increased proliferation of SAT PDGFRβ^+^ adipogenic stem cells in females only.

**Conclusions:**

Sex differences in fibrillar collagen composition and remodelling may promote greater SAT expansion in females by facilitating adipogenic precursor proliferation and limiting fibroinflammatory changes during obesity.

**Supplementary Information:**

The online version contains supplementary material available at https://doi.org/10.1186/s13293-026-01012-6.

## Introduction

A high capacity for physiological subcutaneous adipose tissue (SAT) expansion protects the body from harmful lipid deposition in the visceral compartment, thus decreasing the risk for metabolic diseases [1–5]. In general, the SAT expandability is higher in pre-menopausal women than in men [6–11]. In mice, the capacity for high-fat diet (HFD)-induced SAT adipogenesis is higher in females than in males; this difference is due to altered fat depot microenvironment as opposed to cell-intrinsic mechanisms [12]. Moreover, female mice are less susceptible to HFD-induced adipose tissue inflammation and metabolic disturbances, even when matched for body weight [13]. One factor that can limit the SAT expandability is the stiffness of the extracellular collagen fibers [14–16]. Adipose tissue collagen is found in the extracellular matrix (ECM) (primarily collagen types 1, 3 and 6) and in septa (primarily collagen types 1 and 3) that compartmentalize the adipose tissue into smaller units [17, 18]. These collagenous structures undergo constant remodelling to allow adipocytes and other adipose tissue-resident cells to change their shape and/or function and thereby adapt to nutritional cues [19, 20]. Collagen types 1, 3 and 6 are highly abundant in adipose tissue. Collagen types 1 and 3 are fibril-forming collagens, whereas collagen type 6 forms microfibrils. The ratio between collagen types 1 and 3 contributes to tissue stiffness [21] and varies considerably between tissues [22]. However, a physiological reference ratio has, to our knowledge, not been established for SAT. We and others have shown that acute SAT expansion following 1-week HFD-feeding in male mice is linked to a dramatic loss of collagen type 1 [17, 23, 24], presumably allowing the adipocytes to grow without mechanical constraints. Conversely, upregulation of collagen type 1 leading to a high collagen type 1-to-3 ratio leads to tissue stiffening and is linked to e.g. myocardial fibrosis [25]. Moreover, metabolically unhealthy SAT also shows an upregulation of other factors that increase the rigidity of the ECM such as the collagen-cross-linking enzyme lysyl oxidase (LOX) [26]. Previous research suggest that men are more prone to fibrosis [27], and collagen expression in mouse skin shows sexual dimorphism with lower collagen type 1 expression in females [28].

Here, we investigated sex differences in SAT fibrillar collagen content and composition and their functional implications. We found a higher collagen type 1-to-3 ratio in male than female SAT in both mice and humans. A lower, female-like collagen type 1-to-3 ratio promoted preadipocyte proliferation in vitro, consistent with increased adipocyte precursor proliferation in female SAT during acute expansion. Moreover, female mice exhibited fewer fibroinflammatory changes and better metabolic outcomes during obesity. Together, our findings suggest that sex differences in fibrillar collagen composition and remodelling contribute to the greater capacity for adaptive SAT expansion in females.

## Results

### Healthier SAT expansion in females than in males

**Fig. 1:**
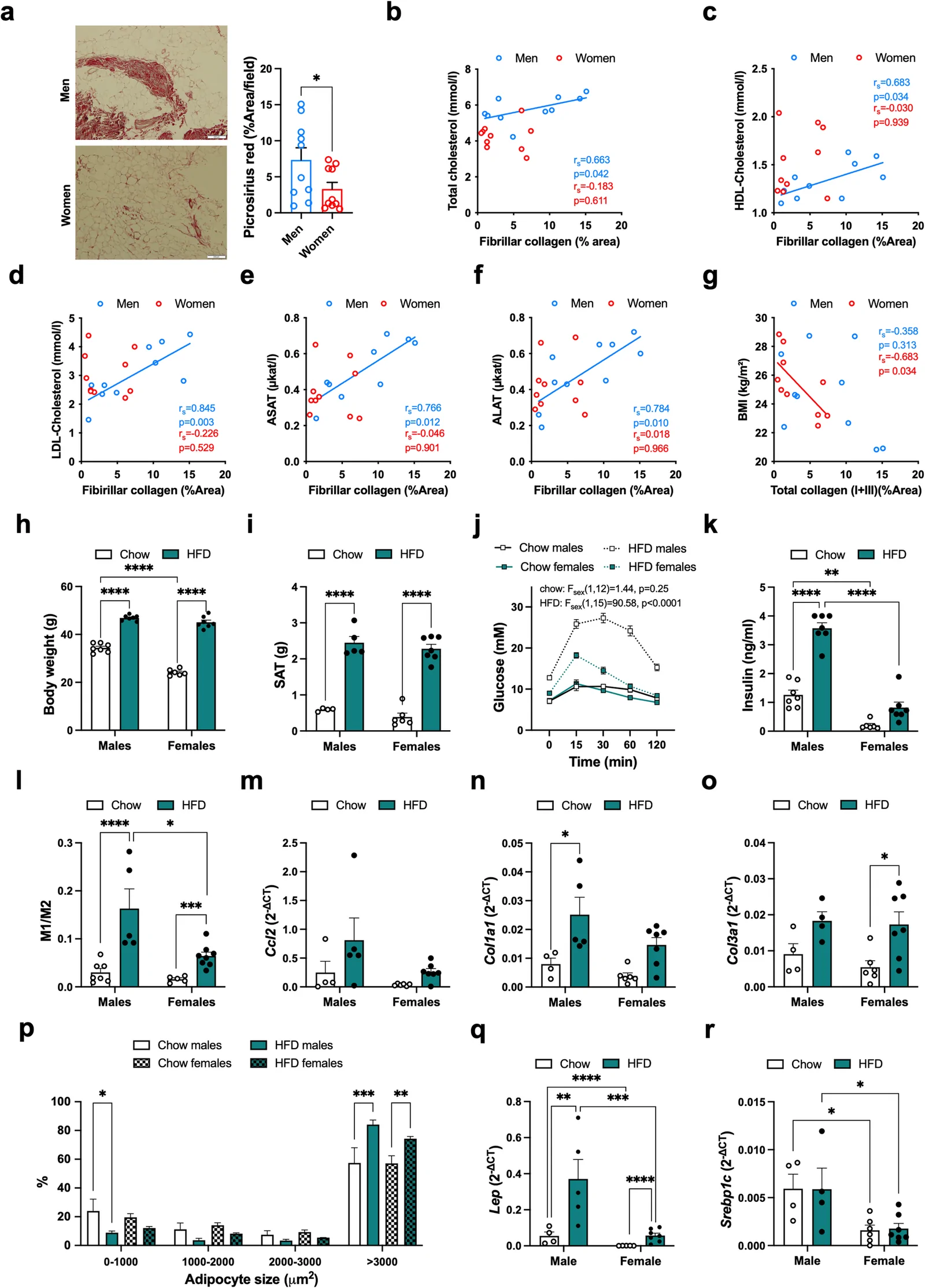
Sex differences in subcutaneous adipose tissue fibrillar collagen, metabolic health and remodelling in humans and mice. (**a**) Representative images and quantification of picrosirius red staining of subcutaneous adipose tissue (SAT) from BMI-matched men and women (*n* = 10/sex; scale bar, 200 μm; ≥3 images per sample). Correlations between SAT fibrillar collagen content and (**b**) total cholesterol, (**c**) HDL cholesterol, (**d**) LDL cholesterol, (**e**) ASAT, (**f**) ALAT and (**g**) BMI in men and women. (**h–r**) Male and female C57BL/6 N mice were fed chow or a high-fat diet (HFD) until the HFD-fed groups reached an average body weight > 45 g (*n* = 6–7/group). Effects of HFD on (**h**) body weight, (**i**) SAT weight, (**j**) oral glucose tolerance and (**k**) fasting insulin levels. (**l**) Ratio of pro-inflammatory (M1-like) to anti-inflammatory (M2-like) macrophages. SAT mRNA expression of (**m**) *Ccl2*, (**n**) *Col1a1* and (**o**) *Col3a1*. (**p**) SAT adipocyte size distribution. SAT mRNA expression of (**q**) *Lep* and (**r**) *Srebp1c*. Differences between men and women in (**a**) were analysed using unpaired Student’s t-test, and associations in (**b-g**) were analysed using Spearman correlation. Mouse data in **h**, **i**, **k**, **l**, **n**, **o**, **q**, and **r** were analysed by two-way ANOVA followed by Šídák’s multiple-comparisons test. Data for the M1/M2 macrophage ratio (**l**), and *Lep* expression (**q**) were log-transformed before analysis. Oral glucose tolerance (**j**) and adipocyte size distribution (**p**) were analysed by two-way repeated-measures ANOVA, with time and adipocyte size category, respectively, as the repeated-measures factor, followed by Šídák’s multiple-comparisons test for prespecified comparisons. As *Ccl2* expression (**m**) remained non-normally distributed after log transformation, differences between chow- and HFD-fed mice within each sex were analysed using the Mann-Whitney U test. *p < 0.05, **p < 0.01, ***p < 0.001, ****p < 0.0001

To study sex differences in SAT expansion in relationship to metabolic health, we stained SAT samples from a cohort of men and women (*n* = 10/sex) with similar age and BMI (20.8–28.8 kg/m^2^) for fibrillar collagen (Table 1). Men had higher total cholesterol levels and 50% higher SAT fibrillar collagen positive areas with a trend (*p* = 0.08) towards higher fibrosis score (Fig. 1a; Table 1). The SAT fibrillar collagen area positively correlated with cholesterol levels (total, HDL and LDL) as well as with liver enzymes ASAT and ALAT in men but not in women (Fig. 1b-f). Similar trends were seen for triglyceride, fatty acid and insulin levels (Table 1). In contrast, SAT fibrillar collagen area was negatively correlated with BMI in women but not in men (Fig. 1g). These data suggest adaptive SAT expansion in this cohort of women, characterized by reduced fibrillar collagen that may permit growth, consistent with observations in healthy mouse models [17, 23, 24]. In contrast, the SAT collagen content in the men appears more consistent with pathological SAT expansion.

**Table 1:** Subject characteristics and correlation with SAT fibrillar collagen levels

Variable	Men			Women		
	Mean ± SEM	Spearman *r* (vs. SAT collagen)	*p*	Mean ± SEM	Spearman *r* (vs. SAT collagen)	*p*
Age (years)	43.10 ± 2.9	-0.923	**0.0003**	40.40 ± 3.5	0.207	0.5636
BW (kg)	81.70 ± 3.3	-0.024	0.9522	71.90 ± 1.4*	-0.390	0.2627
Height (m)	1.82 ± 0.0	0.349	0.3218	1.69 ± 0.0***	0.505	0.1386
BMI (kg/m²)	24.64 ± 0.9	-0.358	0.3129	25.39 ± 0.7	-0.683	**0.0341**
HbA1c (%)	5.36 ± 0.1	0.012	0.9793	5.28 ± 0.1	-0.183	0.6089
FPG (mM)	5.31 ± 0.1	0.006	0.9924	5.00 ± 0.1	0.1281	0.7251
FPI (pM)	24.41 ± 3.8	0.511	0.1351	28.14 ± 4.5	-0.207	0.5636
HOMA-IR	0.96 ± 0.2	0.442	0.2044	1.05 ± 0.2	-0.207	0.5636
TC (mM)	5.77 ± 0.2	0.663	**0.0415**	4.24 ± 0.2***	-0.183	0.6110
HDL (mM)	1.34 ± 0.1	0.683	**0.0342**	1.53 ± 0.1	-0.030	0.9390
LDL (mM)	3.04 ± 0.3	0.845	**0.0032**	3.04 ± 0.2	-0.226	0.5292
TG (mM)	1.41 ± 0.1	0.612	0.0667	1.15 ± 0.2	0.079	0.8310
Ty-G index	8.66 ± 0.1	0.539	0.1139	8.32 ± 0.2	0.085	0.8167
FFAs (mM)	0.22 ± 0.1	0.571	0.0893	0.18 ± 0.0	-0.488	0.1544
CRP (mg/dL)	1.82 ± 0.5	0.479	0.1663	1.19 ± 0.3	-0.293	0.4087
ASAT (µkat/L)	0.50 ± 0.1	0.766	**0.0127**	0.39 ± 0.0	-0.046	0.9014
ALAT (µkat/L)	0.50 ± 0.1	0.784	**0.0098**	0.43 ± 0.0	0.018	0.9661
gGT (µkat/L)	0.43 ± 0.1	0.139	0.7072	0.50 ± 0.1	0.291	0.4090
Fibrosis score	1.20 ± 0.2	0.740	**0.0197**	0.50 ± 0.2	0.851	**0.0024**
Av. adipo. size (µm^2^)	10135.56 ± 1002.5	-0.442	0.2044	9822.38 ± 1039.8	-0.396	0.2562

Values are mean ± SEM. Correlations were assessed using Spearman’s rank correlation coefficient. Statistically significant associations (*p* < 0.05) are shown in bold. *p < 0.05, **p < 0.01, ***p < 0.001 indicate significantly different from men. Abbreviations: BW = Body weight, BMI = body mass index, FPG = Fasting plasma glucose, FPI = Fasting plasma insulin, HOMA-IR = Homeostatic Model Assessment of Insulin Resistance, Av. adipo. size = Average adipocyte size, TC = Total cholesterol, HDL =​​ ​High-density lipoprotein cholesterol, LDL​ = Low-density lipoprotein cholesterol, TG = Triglycerides, TyG = Triglyceride-Glucose index, FFAs = Free fatty acids, CRP​ =​ C-reactive protein, ASAT = Aspartate Aminotransferase, ALAT =​ Alanine aminotransferase, gGT =​ gamma-glutamyl transferase

To further study this sex difference, we challenged mice with HFD until they reached > 40 g of body weight (Fig. 1h) and similar SAT weight (Fig. 1i). HFD-induced obesity was associated with worse glucose tolerance (Fig. 1j), but this effect was much more dramatic in male mice, in accordance with previous reports [29, 30]. Insulin levels and HOMA index were much lower in females than in males, both in chow controls and in the HFD-induced obese group, again suggesting higher insulin sensitivity in females versus male mice (Fig. 1k and Supplementary Figure S1a). The SAT ratio between M1- and M2-like macrophages increased in obese mice, but this increase was more pronounced in males (Fig. 1l) along with increased *Ccl2* expression (Fig. 1m), suggesting a higher degree of inflammation. Moreover, males preferentially upregulated *Col1a1* while females had a more distinct upregulation of *Col3a1* upon prolonged HFD challenge (Fig. 1n, o). HFD-induced obesity was linked to an increased proportion of large adipocytes in both sexes with a trend for more pronounced increase in males and the proportion of smaller adipocytes was reduced in males only (Fig. 1p). In addition, *Lep* and *Srebp1c* expression was higher in HFD-fed males than in diet-matched females (Fig. 1q, r), while obese females had lower lipid accumulation in the liver compared to the males (Supplementary Figure S1b). Thus, both women and female mice better preserve SAT function during expansion, as indicated by a lower degree of fibroinflammatory changes than in males.

### Higher collagen type 1-to-3 ratio in male SAT

**Fig. 2:**
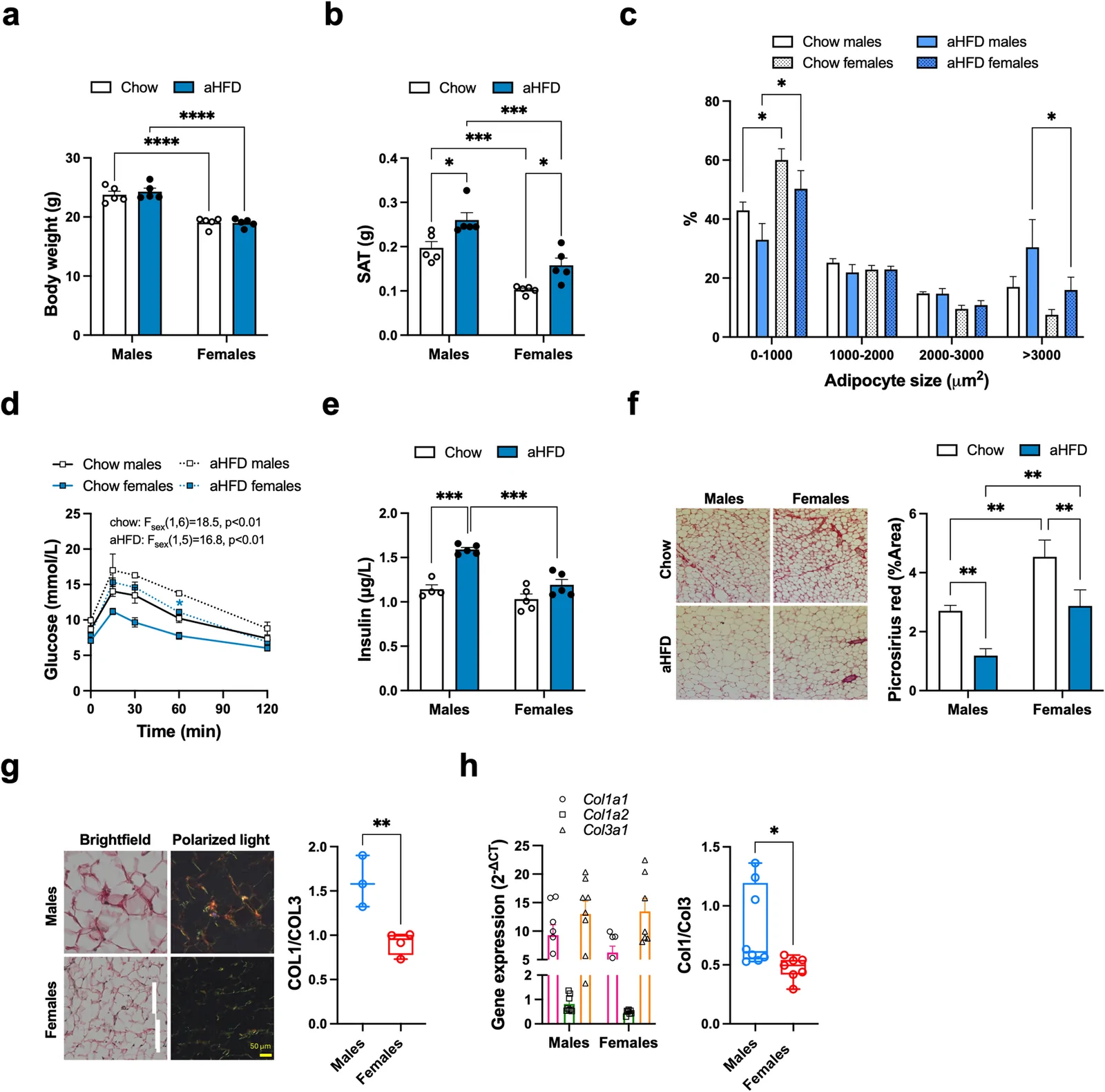
SAT expansion and fibrillar collagen remodelling in male and female mice during acute HFD challenge. Male and female C57BL/6 N mice were placed on chow or acute HFD for one week (aHFD). Effect of aHFD on (**a**) body weight and (**b**) SAT weight. (**c**) Adipocyte size distribution in SAT of chow and aHFD fed male and female 9-week old mice (*n* = 4–5/group). Oral glucose tolerance test (**d**) and fasted insulin levels (**e**) of chow and aHFD fed male and female mice (*n* = 4–5/group). (**f**) Representative image and quantification of picrosirius red staining of male and female chow and aHFD fed SAT (*n* = 4–5/group). Scale bar 100 μm. At least 3 pictures were taken per sample. (**g**) Representative images and collagen type 1-to-3 ratio in SAT between chow-fed males and females measured with polarised light microscopy (*n* = 3–4/group, scale bar 50 μm, at least 3 pictures were taken per sample). (**h**) SAT *Col1a1*, *Col1a2*, and *Col3a1* mRNA levels, and (*Col1a1* + *Col1a2*)-to-*Col3a1*-mRNA ratio in 8-week-old male and female mice (*n* = 7–8/group). Data are presented as mean ± SEM. The effects of diet and sex in **a**-**f** were analysed by two-way ANOVA with Šídák’s multiple-comparisons test (adipocyte size and time as repeated measures factor for respectively **c** and **d**). Differences between two groups in **g** and **h** were analysed by t-test after log-transformation. *p < 0.05 **p < 0.01 ***p < 0.001 ****p < 0.0001

**Fig. 3:**
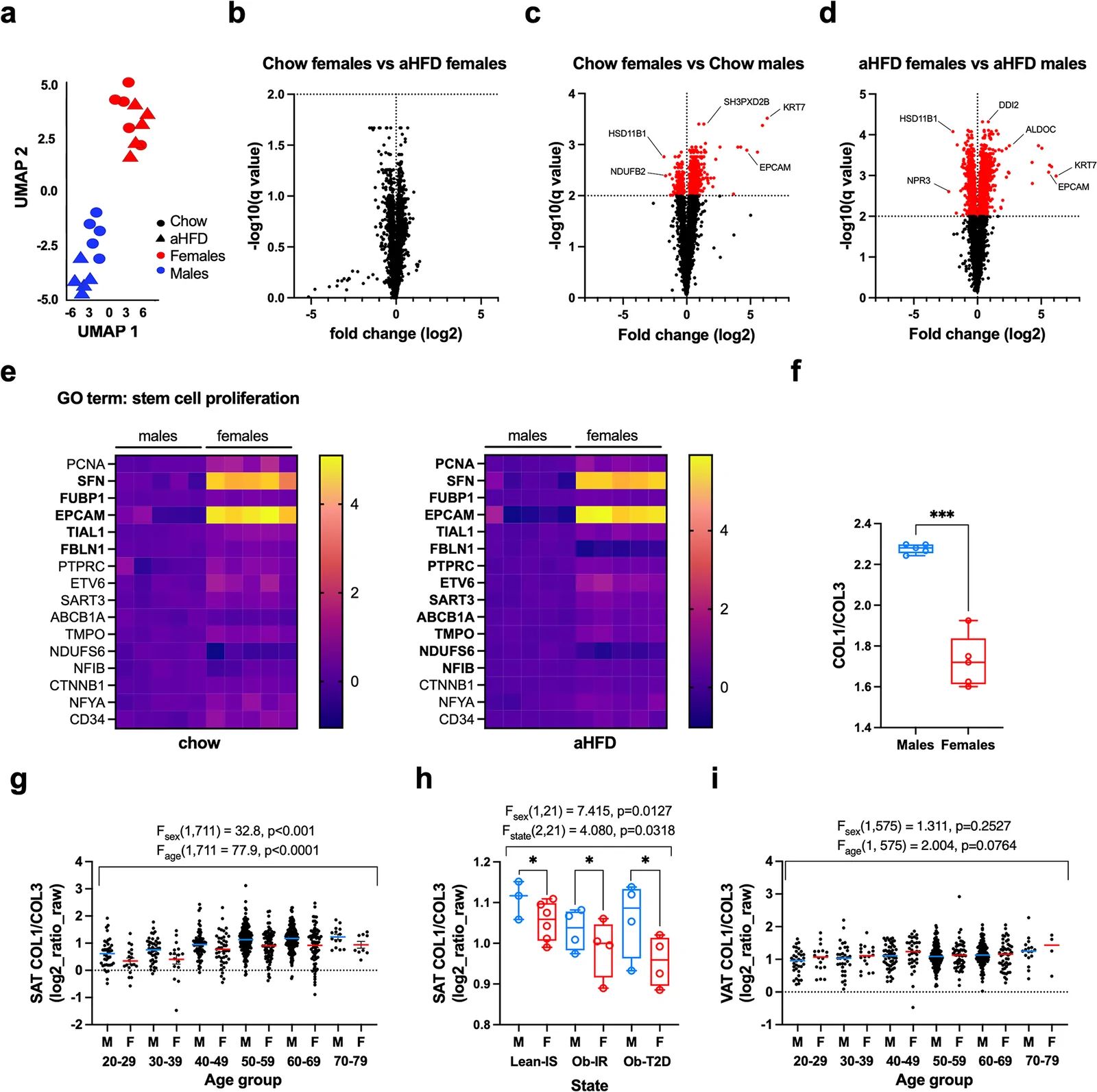
Sex differences in SAT proteome and fibrillar collagen composition in mice and humans. (**a**) Dimensionality reduction with uniform manifold approximation and projection (UMAP). Volcano plots representing the differentially abundant SAT proteins between (**b**) chow and aHFD females, (**c**) chow females and males, and (**d**) aHFD females and males detected and quantified by LC-MS/MS using TMT multiplexing. (**e**) Heatmap of proteins involved in GO term: stem cell proliferation in (left) chow females vs. chow males and (right) aHFD females vs. aHFD males. Heatmap colour indicates protein abundance. Proteins that are significantly different between the groups are in bold (*p* < 0.01). (**f**) (COL1A1 + COL1A2)-to-COL3A1-protein abundance ratio in chow males and females. (*COL1A1* + *COL1A2*)-to-*COL3A1*-mRNA log2 ratio in human SAT across (**g**) different age groups and in (**h**) subjects without obesity and insulin resistance (Lean-IS), with obesity and insulin resistance (Ob-IR), and with obesity and type 2 diabetes (Ob-T2D). (**i**) (*COL1A1* + *COL1A2*)-to-*COL3A1*-mRNA log2 ratio in human VAT across different age groups. Data in **f**-**i** are presented as mean ± SEM. aHFD; acute HFD for one week, *n* = 5/group. Multiple unpaired *t-*test with Welch correction, and false discovery rate set to 1% cut-off (q value above 2 is significant). Differences between the two groups in **f** were analysed by unpaired Student’s t-test after log-transformation. The effects of age and sex in **g** and **i** were analysed with linear regression. The effects of state and sex in **h** were analysed by two-way ANOVA with Šídák’s multiple-comparisons test. *p < 0.05 **p < 0.01 ***p < 0.001 ****p < 0.0001

Our earlier work shows that SAT expansion in young (8-week old) male mice in response to acute 1-week HFD (aHFD) challenge is linked to selective loss of collagen type 1 [17]. Here, we sought to study whether there is a sex difference in this regard. We found that female mice on chow displayed a lower body and SAT weight than males, and this difference between sexes was kept also after challenge with aHFD (Fig. 2a-b). The increased SAT weight in male mice was associated with a lower abundance of small adipocytes compared to females (Fig. 2c). In aHFD challenged mice, males displayed an increased abundance of larger adipocytes compared to females (Fig. 2c). Glucose tolerance was better in females but was equally aggravated following aHFD in both sexes (Fig. 2d, Supplementary Figure S1c). However, insulin level and HOMA index in aHFD females were lower than those of aHFD males, indicating higher insulin sensitivity (Fig. 2e, Supplementary Figure S1d). As expected from the lower SAT weight, females on chow had a higher abundance of fibrillar collagen in SAT than males as judged by picrosirius red staining (Fig. 2f). However, analysis under polarizing light suggests that the collagen type 1-to-3 ratio is different with a higher ratio in male than in female SAT (Fig. 2g). This difference was also apparent at gene expression level (Fig. 2h), like in the older HFD-induced obese mice (Fig. 1n, o). Next, we compared SAT proteome of male and female mice placed on chow or aHFD. The male SAT proteome has been published (21) and now we focused on differences compared to the female proteome that were run with the same batch control to enable comparisons between the data sets. Unlike male SAT that showed a strong response to aHFD [24], the female SAT proteome did not alter upon aHFD, as UMAP analysis clearly showed grouping of female samples regardless of the diet (Fig. 3a). This was confirmed with direct comparison of chow and aHFD female SAT; we found no significant difference in the proteome under the same settings (FDR of 1%, Fig. 3b). Contrary, female proteome was distinct to their diet-matched male counterparts (Fig. 3c, d). Interestingly, some proteins were differentially regulated regardless of the diet, indicating the effect of sex. One of the most upregulated proteins in females was Epithelial cell adhesion molecule (EPCAM) which is involved in stem cell proliferation [31]. 11β-Hydroxysteroid dehydrogenase type 1 (HSD11B1), protein whose overexpression is associated with insulin resistance and central obesity [32], was on the other hand significantly downregulated in females compared to males (Fig. 3c, d). Pathway enrichment analysis indicated increased stem cell proliferation in females compared to males on chow and this difference was enhanced following aHFD (Fig. 3e). The COL1-to-COL3 protein abundance ratio was ~ 30% higher in males than in females (Fig. 3f), consistent with our earlier findings (Fig. 2g, h). As for other proteins related to extracellular matrix structure, for example, the pericellular collagen type 6 proteins COL6A1 and COL6A2, the matrix-organizing collagens COL12A1 and COL18A1, and remodeling proteins such as FN1 and MMP2 were more abundant in female than in male SAT (Supplementary Table S1). In conclusion, proteomic analysis supports previous findings that female SAT is more adapted and/or resilient to HFD-induced changes.

To test whether a similar sex difference in SAT collagen type 1-to-3 ratio is apparent in humans, we analysed the COL1A1, 1A2, and 3A1 gene expression in SAT and VAT from the adult Genotype Tissue Expression (GTEx) Project and SAT from the MD-Lipolysis study that compared individuals with or without obesity, insulin resistance and type 2 diabetes [33]. Indeed, men displayed about 30% higher SAT collagen type 1-to-3 ratio than women across the age groups (Fig. 3g) and metabolic states (Fig. 3h). In contrast, this sex difference was not observed in visceral omental adipose tissue (Fig. 3i).

### Lower collagen type 1-to-3 ratio is more permissive to preadipocyte proliferation

**Fig. 4:**
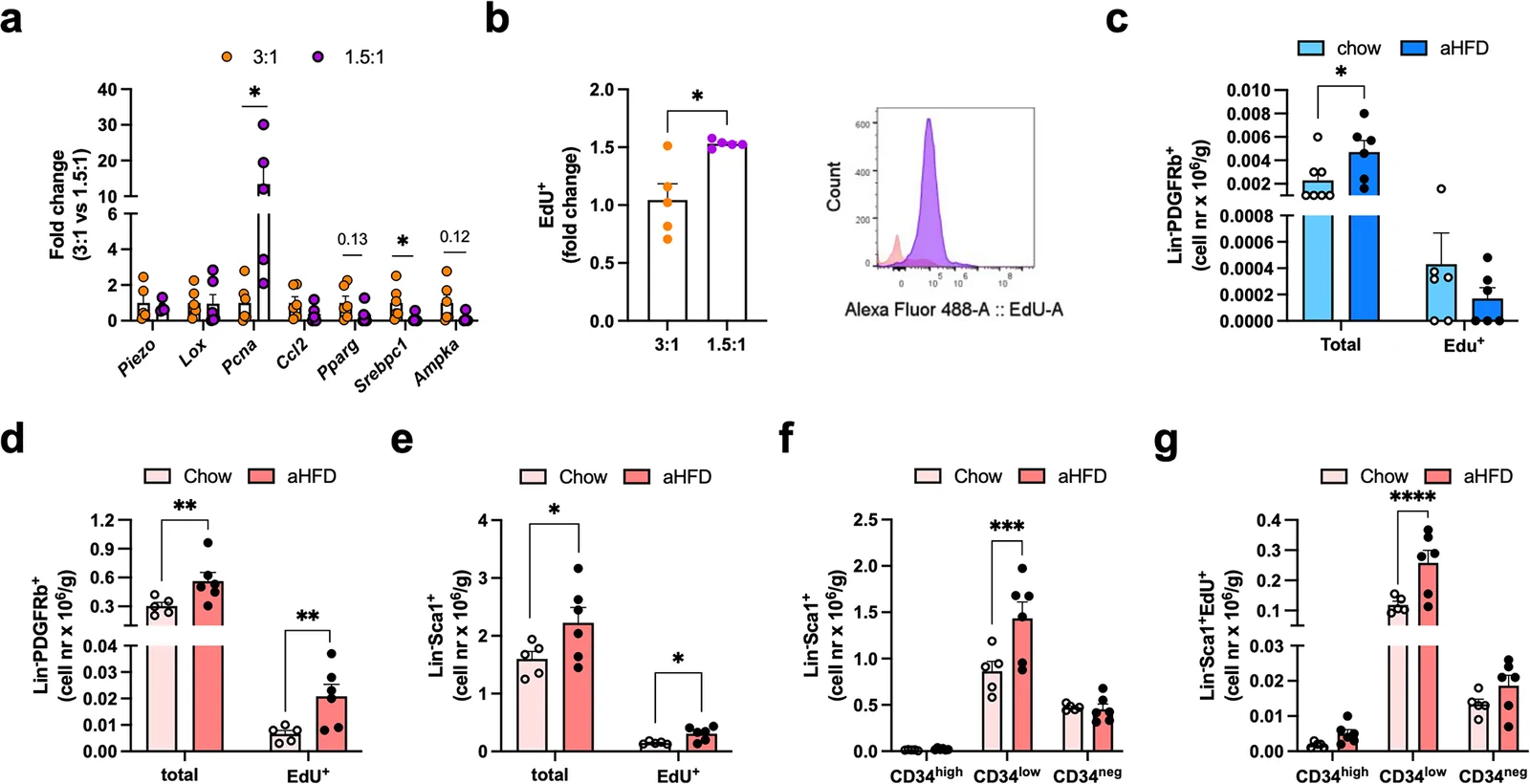
Stem cell proliferation is increased in female-like gels and female SAT upon aHFD challenge. (**a**) Gene expression analysis of 3T3-L1 pre-adipocytes seeded in male-like (collagen 1 to 3 ratio 3:1) and female-like (collagen 1 to 3 ratio 1.5:1) collagen gels for 24 h (*n* = 4–6/group). Data are expressed as relative to male-like gels. (**b)** Proliferation of 3T3-L1 pre-adipocytes seeded in male- and female-like collagen gels as assessed by %Edu^+^ cells out of total single live cells (*n* = 5/group). Two experiments were combined and data are therefore expressed as fold change to male-like collagen gels. Number of total and proliferating CD45^−^CD31^−^(Lin^−^)PDGFRb^+^ stem cells per gram of SAT in (**c**) chow and aHFD males and (**d**) chow and aHFD females (*n* = 6–7/group). (**e**) Number of total and proliferating Lin^−^Sca1^+^ stem cells per gram of SAT in chow and aHFD females (*n* = 5–6/group). Number of (**f**) total and (**g**) proliferating subsets of Sca1 stem cells per gram of SAT in chow and aHFD females (*n* = 5–6/group). CD34^high^ = high lipid turnover progenitors; CD34^low^ = low lipid turnover progenitors; CD34^neg^ = beige adipocyte progenitors; aHFD; acute HFD for one week. Data are presented as mean ± SEM. Differences between the groups in **a-b** were analysed by unpaired Student’s t-test, and in **c-g** with two-way ANOVA with Šídák’s multiple-comparisons test. Flow cytometry data in **d** and **e** were log transformed before analysis. *p < 0.05 **p < 0.01 ***p < 0.001

Since we have consistently found SAT differences in the collagen type 1-to-3 ratio between male and female mice and in humans, we decided to test for functional implication of such difference. To this end, we prepared collagen gels with male-like (3:1) and female-like (1.5:1) collagen type 1-to-3 ratios, reflecting the approximately 2-fold sex difference observed by polarized light microscopy (Fig. 2g). The total fibrillar collagen content was kept constant between gels. Guided by our proteomics data, we analysed markers of proliferation, adipogenesis, and adipocyte functionality after 24 h of culture. We found a rather dramatic upregulation of Proliferating cell nuclear antigen (*Pcna*) in pre-adipocytes grown in female-like gels compared to male-like gels (Fig. 4a). Pre-adipocytes in female-like gels also showed reduced expression of *Srebpc1* with similar trends for *Pparg* and *Ampka* whereas the expression of the ‘mechanosensor’ *Piezo*, *Lox*, and the chemokine *Ccl2* were similar between groups (Fig. 4a). Proliferation as assessed by Edu-incorporation (Edu% of single live cells) was also increased in female-like gels (Fig. 4b). These data prompted us to compare the SAT stem cell abundance and proliferation in female and male mice following aHFD challenge. We have previously shown that the proliferation of SAT Sca1^+^ stem cells increase in male mice [24]. However, the more adipocyte-committed, PDGFRβ^+^ stem cells did not proliferate at a higher rate as judged by no difference in EdU incorporation in male SAT (Fig. 4c). This was contrasted by female SAT, where both Sca1^+^ and PDGFRβ^+^ stem cells displayed increased proliferation compared to chow controls (Fig. 4d, e). Of note, proliferation of Sca1^+^ stem cells was confined to CD34^low^ subset (Fig. 4f, g) which in human SAT gives rise to low lipid turnover adipocytes [34]. Together, these findings suggest that female-like fibrillar collagen composition stimulates stem cell proliferation while male-like composition may be more permissive for lipid accumulation and adipogenic differentiation.

## Discussion

Here we show that female and male SAT display dimorphism in the fibrillar collagen composition with males showing higher collagen type 1-to-3 ratios compared to females. We furthermore show that matrix prepared with a lower, female-like, collagen type 1-to-3 ratio is more permissive to stem cell proliferation. This aligns with our findings in vivo where we observed elevated stem cell proliferation in female SAT following aHFD. In addition, the female SAT proteome shows upregulation of several proteins involved in proliferation. Finally, male mice exhibited more pronounced obesity-induced fibroinflammatory changes in SAT and worse metabolic outcomes compared to equally obese females, and similar associations were observed in our human cohort. Thus, our data are in accordance with a greater capacity for adaptive SAT expansion in females.

Our findings support the notion that the fibrillar collagen content in SAT in a healthy setting reflects the degree of adiposity rather than fibrosis [35]. However, the composition of fibrillar collagens is likely to play a role; collagen type 1 forms relatively stiff fibres that gives tensile strength while collagen type 3 produces thin elastic fibres [36]. Thus, a physiological collagen type 1-to-3 ratio provides optimal tissue support while preserving elasticity and could thus play an important role in tissue functionality. Indeed, increased collagen type 1-to-3 ratio is associated with elevated myocardial stiffness [37], increased scarring after breast conserving surgery [38]. However, aging has been reported to exert tissue-specific effect on the collagen type 1-to-3 ratio in rodents; in lungs and heart the ratio decreases while it increases in the skin [39, 40]. Adipose tissue fibrosis is manifested as large deposits of collagen type 1 and 3 in tissue parenchyma and pericellular fibrotic deposition [18], but the SAT collagen composition in healthy males and females has not been studied. Previous research shows that in mouse skin, male mice exhibit higher *Col1a1* and *Col1a2* mRNA levels compared to females, with levels remaining high in males throughout postnatal development [28]. Our data show that SAT display a similar sex difference in both mice and humans. In human SAT, we also observe that the collagen type 1-to-3 ratio increases with age, at least at the transcriptional level. This may have functional implications as our in vitro data suggest that a lower collagen type 1-to-3 ratio is more permissive to preadipocyte proliferation. Conversely, a higher male-like collagen type 1-to-3 ratio increased *Srebp1c* expression, which was also higher in male mouse SAT. SREBP1c is induced during adipocyte differentiation and regulates lipogenic gene expression and lipid accumulation [41]. Thus, the altered *Srebp1c* expression may indicate differences in adipogenic/lipogenic programming associated with fibrillar collagen composition. However, whether ECM stiffness directly regulates SREBP1c in this context remains to be established.

Adipose tissue expansion through increasing adipocyte number (hyperplasia) rather than size (hypertrophy) is considered a more favourable mode of adipose tissue expansion, while adipocyte hypertrophy is generally linked to insulin resistance [42, 43]. Ability to increase the pool of adipose tissue stem cells by proliferation supports hyperplastic growth, and we show that subsets of SAT stem cells in female mice proliferate more than male ones during aHFD challenge. This finding is in accordance with previous research [12]. However, SAT expansion in mice is primarily driven by hypertrophy [43, 44], possibly with a higher contribution of adipogenesis in females [12]. Notably, we only observe subtle differences in adipocyte size in between male and female SAT of equal size, suggesting similar adipogenesis, possibly with differences in the adipocyte turnover. Yet the better glucose tolerance and lower hepatic lipid levels is consistent with improved adipose tissue storage function in female mice. One possible explanation is that female SAT microenvironment such as the ECM composition allows adipocytes to grow without losing as much of their metabolic function. The lower leptin expression in female SAT is in line with this idea; leptin expression is typically correlated with adipocyte size and is upregulated by hypoxia and TNFα [17, 45]. It is thus tempting to speculate that male adipocytes, despite being of similar size, display increased HIF1α activation, contributing to the enhanced fibroinflammatory changes in male SAT [46]. A similar phenomenon is observed in collagen 6 knockout Ob/Ob mice, where absence of collagen type-6 fibrils leads to improved metabolic phenotype despite larger adipocyte size [15]. Interestingly, adipose tissue ECM from non-diabetic subjects has been shown to rescue metabolic dysfunction in adipocytes from patients with type-2 diabetes, whereas adipose tissue ECM from type-2 diabetic subjects impaired the metabolic function of non-diabetic adipocytes [47].

In the men of our cohort, the SAT fibrillar collagen content correlated strongly with cholesterol and liver enzyme (ALAT and ASAT) levels, with similar trends for triglyceride and fatty acid levels. Interestingly, fibrillar collagen content correlated positively with both LDL- and HDL-cholesterol. Although this may appear counterintuitive, HDL- and LDL-cholesterol were not inversely correlated in these men (r_s_=0.450, *p* = 0.192), and mean HDL-cholesterol levels were within a healthy range. These findings align with earlier SAT findings in Chinese individuals [48] and may reflect fibrosis-associated adipose tissue dysfunction characterized by impaired lipid storage and increased spillover into the circulation [48–51]. However, the increased cholesterol levels in men may also contribute to adipose tissue fibrosis progression via its proinflammatory effects [52, 53].

Limitations of our study include the lack of human SAT protein data and not taking other adipose tissue stiffness contributing factors into account such as LOX-generated cross-links, fiber alignment, and other ECM components e.g. collagen type-6, fibronectin, laminins, elastin and proteoglycans.

In conclusion, our findings indicate that alterations in fibrillar collagen composition are one mechanism through which the ECM regulates adipose tissue function. More research in this area may uncover further mechanistic insights and guide interventions aimed at promoting physiological adipose tissue expansion to maintain metabolic health and prevent cardiometabolic diseases.

## Methods

### Ethics statement

Ethical approvals regarding collection and use of human adipose tissue have been granted by the Ethics Committee of the University of Leipzig (approval numbers: 159-12-21052012 and 017-12ek to Prof. Blüher) and the Regional Ethics Review Board of the University of Gothenburg (Diary number: 844 − 12) and the Swedish Ethical Review Authority (Diary number: 2019–04911) to Prof. Jansson - MD-Lipolysis study. The clinical studies were performed in accordance with the declaration of Helsinki and all subjects gave written informed consent before taking part.

All animal experimental protocols were approved by the Animal Ethics Committee in Gothenburg (2733/20), followed the EU Directive 2010/63/EU, and are reported according to ARRIVE guidelines.

### Human SAT

The data used for the analyses of SAT COL1A1, 1A2, and 3A1 gene expression were obtained from ‘gene_reads_v10_adipose_subcutaneous.gct.gz’ and ‘gene_reads_v10_adipose_visceral.omentum.gct.gz’ from the GTEx Portal (https://www.gtexportal.org/home/) on 10/22/25, and from the MD-Lipolysis cohort (GSE141432) [33].

Paraffin-embedded SAT sections were obtained from fasted healthy women (*n* = 10) and men (*n* = 10) with similar BMI without obesity. The anthropometrics for these subjects are given in Table 1. The biopsies were taken under local anaesthesia.

### Mouse study protocol

Five-week-old C57Bl/6 N male and female mice purchased from Janvier Labs (Le Genest-Saint-Isle, France) were housed at the animal core facility of the Sahlgrenska Academy at the University of Gothenburg. All mice were housed under standard conditions: temperature 24 °C, 12 h light/dark cycle from 7am, and *ad libitum* access to water and chow (Teklad global rodent diet 2016, Inotiv, Madison, WI, USA) or a HFD (HFD, D12492, Research Diets Inc., New Brunswick, NJ, USA).

At the age of eight weeks, mice were randomly assigned to continuation on standard chow or HFD for one week; acute HFD (aHFD). Another group of mice were randomly assigned to continuation on standard chow or HFD until the HFD-group reached at least 40 g. To study proliferation, aHFD mice were injected with 10 mg/kg EdU i.p. (5-ethynyl-2´ -deoxyuridine, Invitrogen, Carlsbad, CA, USA) 24 h before euthanization. Glucose tolerance was measured by a glucose tolerance test: Mice, fasted for four hours, received an oral glucose load (2.5 g/kg body weight of D-(+)-glucose dissolved in water, G8270, Sigma Aldrich, St. Louis, MA, USA). Blood glucose was measured at the tail vein with a glucometer (Contour XT, Bayer, Basel, Switzerland) at baseline and at 15, 30, 60 and 120 min after the glucose load. Serum insulin levels were measured by a commercial ELISA kit (Mouse Insulin ELISA, Mercodia, Uppsala, Sweden) per manufacturer’s instructions. Tissues were used for histological analysis, collected for stromal vascular fraction (SVF) isolation, or snap frozen in liquid nitrogen and kept at -80 °C until further analyses. The number of mice used for each experiment is indicated in the figure legends and varied between 3 and 8. We exclude mice that show signs of illness. The order of measurements and sample processing was balanced across experimental groups.

### Histology

SAT (inguinal white adipose tissue) was fixed in 4% buffered formalin (VWR Chemicals, Stockholm, Sweden) for 48 h, paraffin embedded and cut into 7 μm thick sections. Picrosirius red (Picrosirius Red Stain Kit, Polysciences, Warrington, USA) was used according to manufacturer´s instructions to stain fibrillar collagen in deparaffinized sections. Fibrillar collagen content was quantified as the percentage of tissue area positive for picrosirius red staining. At least three representative images were taken for each mouse/human section using a light microscope (Olympus BX60, Olympus, Tokyo, Japan) equipped with a Plan-Apo 20x/0.8 objective. Adipocyte size distribution was determined in stained tissues using Adiposoft plugin on Fiji (Image J, version 1.54p). Collagen type 1-to-3 ratio in fat was measured with polarised light microscopy.

### Gene expression analysis

SAT mRNA was isolated using ReliaPrep™ RNA Cell Miniprep System (Promega, Madison, WI, USA) and cDNA was prepared using High-capacity RNA-to-DNA kit (Life Technologies, Carlsbad, CA, USA) according to the manufacturer’s instructions. PCR reactions were detected using SYBR Green (Life Technologies) and performed on QuantStudio 7 flex system (Applied Biosystems, Foster City, CA, USA) using the comparative threshold cycle (Ct) method for relative quantification. Beta actin (*Bactin*) and TATA-Box Binding Protein (*Tbp)* were used as endogenous controls for 3T3-L1 cells and SAT respectively. Two samples could not be analyzed due to insufficient RNA quality. Primer sequences are summarized in Supplementary Table S2. Data were analysed in Quant Studio Real time PCR software v1.3 (Thermo Fisher).

### Proteomics sample preparation, LC-MS/MS, and data analysis

SAT samples were prepared as previously described [24]. In short, SAT were homogenized, trypsin digested and filtered to receive purified peptide fragments with masses < 30 kDa. Purified peptides were labelled using Tandem Mass Tag reagents and samples were fractionated into 40 primary fractions by basic reversed-phase chromatography. The enriched phosphopeptide fractions were concatenated into final fractions, evaporated and reconstituted before analysed by LC-MS/MS on an Orbitrap Fusion Tribrid™ mass spectrometer interfaced with an Easy-nLC 1200 liquid chromatography system (Thermo Fisher Scientific). Identification was performed using Proteome Discoverer version 2.4 (Thermo Fisher Scientific). The database search was performed using the Mascot search engine v. 2.5.1 (Matrix Science, London, UK) against the Swissprot mouse database. Only the unique identified peptides were considered for protein quantification. The TMT reporter ions were identified with 3 mmu mass tolerance in the MS2 or MS3 HCD spectra and the TMT reporter S/N values for each sample were normalized within Proteome Discoverer 2.4 on the total peptide amount. The protein abundances were further normalized to the abundance of a reference sample to allow for comparison between runs. The mass spectrometry proteomics data have been deposited to the ProteomeXchange Consortium via the Proteomics Identifications (PRIDE) partner and the Mass Spectrometry Interactive Virtual Environment (MassIVE) repositories with the dataset identifiers PXD043721 and PXD086747​.

### Preadipocyte cell line

Mouse pre-adipocyte cell line 3T3-L1 (ZenBio, Durham, NC, U.S.A.) was maintained in high-glucose DMEM (DMEM, high glucose, Thermo Fisher Scientific, MA, U.S.A) supplemented with 1% penicillin-streptomycin (Penicillin-Streptomycin 10,000 U/mL, Thermo Fisher) and 10% foetal bovine serum (FBS) (HyClone™ Characterized Fetal Bovine Serum, Cytiva, Marlborough, USA). Prior to freeze-down, the 3T3-L1 cells were tested for Mycoplasma contamination using a qPCR-based assay and confirmed to be negative. However, the thawed cells used in the present study were not re-tested.

### Collagen gels

Collagen type I (Cultrex bovine collagen I, R&D Systems, Minneapolis, USA) and collagen type III (Native bovine collagen III, BioRad) were diluted in acetic acid to a concentration of 5 mg/mL. Collagens were neutralized in a 8:1:1 ratio to a final concentration of 4 mg/mL with a solution of 10xPBS 100mM HEPES and a mix of 2.10^3^/µL 3T3-L1 cells in DMEM high glucose (DMEM, high glucose, ThermoFisher) supplemented with 10% FBS (HyClone™ Characterized Fetal Bovine Serum, Cytiva). Collagen gel mixes were then placed in 24-well plates inserts (Falcon™ Cell Culture Inserts 8.0 μm, Corning Inc., Corning, USA) and left to polymerize for 45 min at 37 °C, 5%CO_2_. After polymerization, the plate wells and inserts were filled with 500µL DMEM high glucose 10%FBS each (DMEM, high glucose, Thermo Fisher Scientific, MA, U.S.A). For proliferation measurement, EdU (20 µM, Invitrogen) was added and incubated for 4 h before flow cytometry analysis.

### SVF isolation and flow cytometry

SAT from inguinal fat pads were digested in buffer containing 123 mM NaCl, 5 mM KCl, 5 mM CaCl2 1.2 mM KH2PO4, 1.2 mM MgSO4, 25 mM HEPES, 2 mM glucose, 200 nM adenosine, 1.5% bovine serum albumin (BSA), and 2 mg/ml collagenase type II (Sigma-Aldrich). Samples were incubated at 37 °C with shaking (120 rpm). Collagenase activity was stopped by moving samples to room temperature and adding PBS with 1 mM EGTA (ethylene glycol-bis(β-aminoethyl ether)-N, N,N′,N′-tetraacetic acid). Next, SVF samples were filtered through cell strainers (100 μm) and centrifuged at 500 g for 5 min. Red blood cells were removed by RBC lysis buffer (Biolegend, San Diego, CA, USA) treatment for 2 min at room temperature. Then, cells were washed and resuspended in Dulbecco’s modified eagle medium (DMEM) with 10% FBS and counted on an automatic cell counter (Countess 2, Thermo Fisher). An equal number of cells were aliquoted and washed in PBS before non-specific binding was blocked with anti-mouse CD16/CD32 antibody (Miltenyi Biotech, Bergisch Gladbach, Germany) in PBS with 1% BSA for 15 min. Cells were then stained with fluorescently labelled antibodies for 45 min, at + 4 °C in the dark. All antibodies used for flow cytometry are summarized in Supplementary Table S3. Dapi (Miltenyi) was added as a dead cell marker for non-fixed cells, and LIVE/DEAD™ Fixable Blue Dead Cell Stain Kit (Thermo Fisher) was the equivalent for fixed cells. Click-iT™ Plus EdU Alexa Fluor™ 488 Flow Cytometry Assay Kit (Thermo Fisher) was used to detect EdU. Events (20000) were acquired on FACSCANTO 2 flow cytometer (BD Bioscience, Franklin Lakes, NJ, USA). All fluorophores were compensated with compensation controls, and gates were set with Fluorescence Minus One controls. Cells were gated based on their forward scatter and side scatter appearance, followed by selection of single live cells (Supplementary Figure S2). The flow cytometry data was analysed using FlowJo software version 10.6.0 (FlowJo, Ashland, OR, USA) and FACS Diva software version 7.0 (BD Bioscience). Four samples could not be analyzed due to insufficient number of cells.

### Statistics

Differences in clinical characteristics between women and men were assessed using the Mann-Whitney U test and data are presented as mean ± SEM. Correlation of fibrillar collagen content in SAT and clinical variables were investigated using Spearman analysis. In vivo and in vitro data are shown as individual values and mean *±* SEM.

Gene expression for COL1A1, COL1A2, and COL3A1 was analysed in publicly available datasets after applying a log₂ (raw counts + 1) transformation (pseudocount = 1). The COL1-to-COL3 ratio was defined as log₂[(COL1A1 + COL1A2+0.5)/(COL3A1 + 0.5)]. The effects of age and sex on this ratio were assessed using linear regression, and the effects of sex and metabolic state were tested using two-way ANOVA.

In general, differences between two groups were analysed by Student’s t-test and experiments involving two independent variables were analysed by two-way ANOVA followed by Šídák’s multiple-comparisons test. Normality was assessed using the Anderson-Darling, D’Agostino-Pearson, Shapiro-Wilk and Kolmogorov-Smirnov tests, as applicable. For two-way ANOVA, normality was assessed based on model residuals and Q-Q plots. When the assumption of normality was not met, data were log-transformed and reassessed. For two-group comparisons, data that remained non-normally distributed after transformation were analysed using the Mann-Whitney U test.

Glucose tolerance and adipocyte size data were analysed by two-way repeated-measures ANOVA, with time or adipocyte size category, respectively as the repeated-measures factor followed by Šídák’s multiple-comparisons test for prespecified comparisons. All statistical analyses were done with GraphPad Prism 11.

For the proteomics data analysis, abundances were normalized and log transformed. Differences in protein expression were calculated on log2 fold change. Differentially expressed proteins between males and females were analysed using multiple unpaired t-test, which were corrected for multiple testing using Welch correction, with false discovery rate set to 1%. Proteomic data are presented as log2 fold-change. Differentially abundant proteins were subjected to enrichment analyses in Cytoscape (version 3.8.2) using GO and KEGG ontologies. UMAP analysis was conducted in R Studio (version 4.2.1).

## Supplementary Information

Below is the link to the electronic supplementary material.

Supplementary Material 1: Supplementary Figure S1

Supplementary Material 2: Supplementary Figure S2

Supplementary Material 3: Supplementary Tables S1-S3

## Data Availability

The mass spectrometry proteomics data have been deposited to the ProteomeXchange Consortium. All other data supporting the findings of this study are available within the article. Further information and requests for resources should be directed to the lead author.
