# Supplementary material for: Sex differences in fibrillar collagen composition are linked to subcutaneous adipose tissue expandability and adipocyte precursor proliferation
Source: Biol Sex Differ. 2026 Nov 13;17:181. doi: 10.1186/s13293-026-01012-6 (PMC13760813; doi:10.1186/s13293-026-01012-6)
Supplement: Supplementary file 1 [file 13293_2026_1012_MOESM1_ESM.pdf]

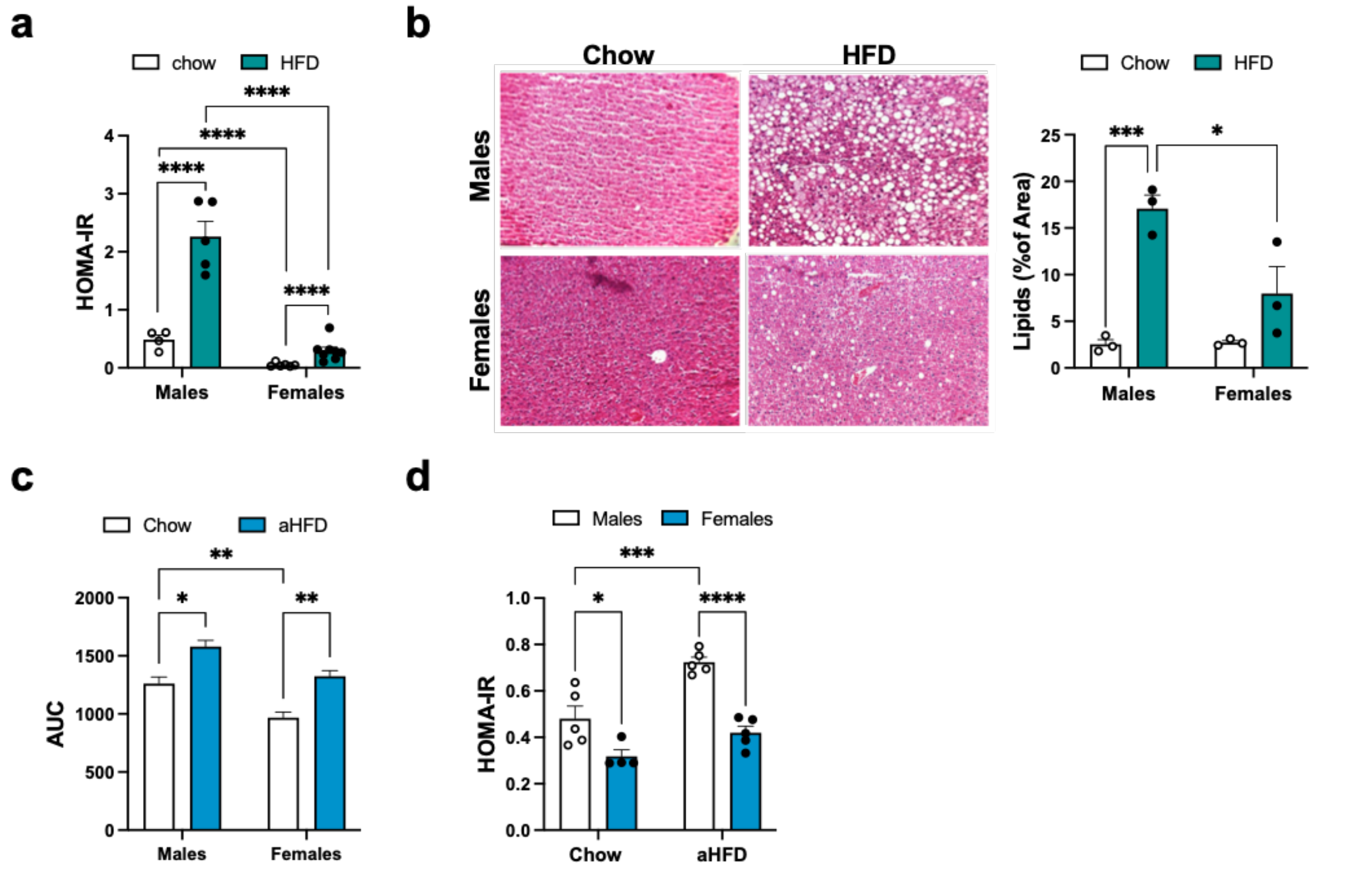

Supplementary Figure 1. (**a**) HOMA-IR index and (**b**) liver lipid accumulation in males and females on chow vs. challenged with HFD-induced obesity. (**c**) OGTT glucose AUC and (**d**) HOMA-IR index in males and females on chow vs. challenged with acute HFD for one week (aHFD). Data are presented as mean ± SEM. Differences between the groups were analysed by two-way ANOVA with Šídák's multiple-comparisons test. Data in **a** were log-transformed before analysis. *p<0.05 **p<0.01 ***p<0.001 ****p<0.0001.
