# Supplementary material for: Sex differences in fibrillar collagen composition are linked to subcutaneous adipose tissue expandability and adipocyte precursor proliferation
Source: Biol Sex Differ. 2026 Nov 13;17:181. doi: 10.1186/s13293-026-01012-6 (PMC13760813; doi:10.1186/s13293-026-01012-6)
Supplement: Supplementary file 2 [file 13293_2026_1012_MOESM2_ESM.pdf]

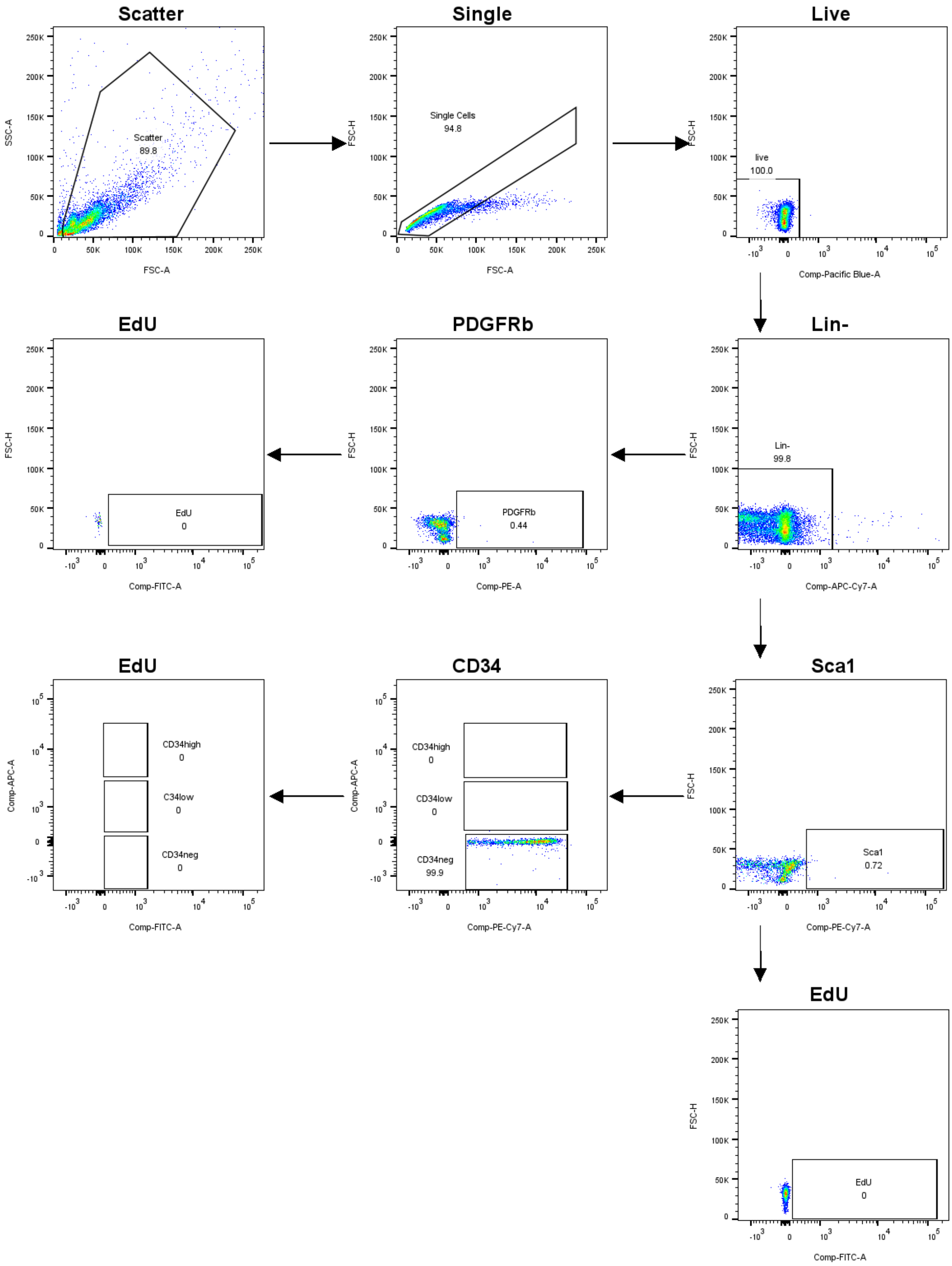

Supplementary Figure 2. Gating strategy used in flow cytometry. All gates were adjusted by Fluorescent Minus One controls.
